# Measurement of Linear Springs’ Stiffness Factor Using Ultrasonic Sensing

**DOI:** 10.3390/s22155878

**Published:** 2022-08-05

**Authors:** Zhongwei Zhang, Xiyan Zhang, Bohui Ma, Mengyao Ding, Bowen Zhu, Dezheng Tong

**Affiliations:** Department of Optical Engineering, College of Optical, Mechanical and Electrical, Zhejiang A & F University, Hangzhou 311300, China

**Keywords:** ultrasonic wave, Jolly’s scale, Hooke’s law, stiffness factor, shape variable

## Abstract

We designed an ultrasonic testing instrument that consisted of a single-chip microcomputer module, a digital display module, and an ultrasonic sensor module, which conveniently eliminated the troubles faced by the traditional Jolly’s scale. For comparison purpose, three linear springs’ stiffness factors were measured by Jolly’s scale and by our ultrasonic testing instrument. We found that our instrument could more conveniently and in real time display the distance values between the ultrasonic ranging module and the horizontal bottom plate when loading different weights. By processing these distance data, we found that our instrument was more convenient for obtaining the linear springs’ stiffness factors and that the results were more accurate than those of Jolly’s scale. This study verified that our instrument can accurately realize the performance of Jolly’s scale under diverse temperatures and humidity levels with high data reliability and perfect stability.

## 1. Introduction

The linear spring stiffness factor *k*, also known as the stubbornness coefficient, is an important property that indicates the ability of the linear spring to resist deformation [1,2,3]. Being a kind of elastic object that stores energy, linear springs are widely used in many fields, such as furniture, architecture, machinery, and electronics [4,5,6,7,8]. Depending on the purpose for their usage, different types of linear springs should have different stiffness factors.

Generally, *k* is measured by Jolly’s scale [9] and then calculated based on Hooke’s law [10,11,12]. However, when using the Jolly’s scale, it is necessary to first “align the three lines” (note: the first line L_1_ is on the surface of the glass tube, the second line L_2_ is on the surface of the mirror, and the third line L_3_ is a reflection image of L_1_ in the mirror). Next, the readout on the Vernier Caliper must be manually identified after each addition of equal weight, and the measurement results must be evaluated. These will incur artificial accidental errors and lead to inaccurate measurement results.

The term ultrasonic wave refers to sound waves with frequencies higher than 20 kHz, and common ultrasonic frequencies range from tens of kHz to tens of MHz. It is a type of mechanical wave that has the advantages of a strong penetrating force, good directionality, and easy access to concentrated sound energy [13,14,15,16]. Due to its advantages of non-contact measurement, low cost, easy operation, and rapid measurement, it has been widely used in industrial measurements, safety warnings, scientific obstacle avoidance by robots, and ultrasonic motors [17,18,19,20,21,22,23]. Particularly in distance measurement, as researchers continue to explore and further expand the ultrasonic ranging technology [24,25,26,27,28], measurement accuracy and the stability of ultrasonic ranging are more suitable for industrial control and high-level instruments.

After searching on the *Web of Science*, we found very few studies on the measurement of the linear spring stiffness factor using ultrasonic sensing, and there was no one instrument using ultrasonic sensing to measure the linear spring stiffness factor. In this study, we used an ultrasonic testing instrument that consisted of a single-chip microcomputer module, a digital display module, and an ultrasonic sensor module to measure displacement. For comparison purposes, three linear springs’ stiffness factors were measured by Jolly’s scale and by the ultrasonic testing instrument. We found that our ultrasonic testing instrument could more conveniently eliminate the troubles faced by the traditional Jolly’s scale and displayed the distance values in real time between the ultrasonic ranging module and horizontal bottom plate when loading different weights. By processing these distance data, we found that our ultrasonic testing instrument was more convenient for obtaining the linear springs’ stiffness factors and that the measurement results were more accurate than those of Jolly’s scale. This study verified that our instrument can accurately realize the performance of Jolly’s scale under diverse temperatures and humidity levels and also excelled in other quantitative ranging measurements.

## 2. Experimental Method

### 2.1. Linear Spring Stiffness Factor k

The linear spring stiffness factor *k* complies with Hooke’s law as follows:

(1) F=k⋅Δx

In Equation (1), F is the elastic force received by the linear spring, and Δx is the deformation quantity of the spring once it is deformed.

In general, we can find the linear spring stiffness factor *k* by following three steps with our ultrasonic testing instrument. First, the weights of different masses are applied to the oscillator to produce a series of forces of known magnitude. Next, the ultrasonic sensor module measures the corresponding deformations of the spring. Finally, in line with the successive differentiation method, the linear spring stiffness factor *k* can easily be obtained from Equation (1).

### 2.2. Device Composition

**Figure 1:**
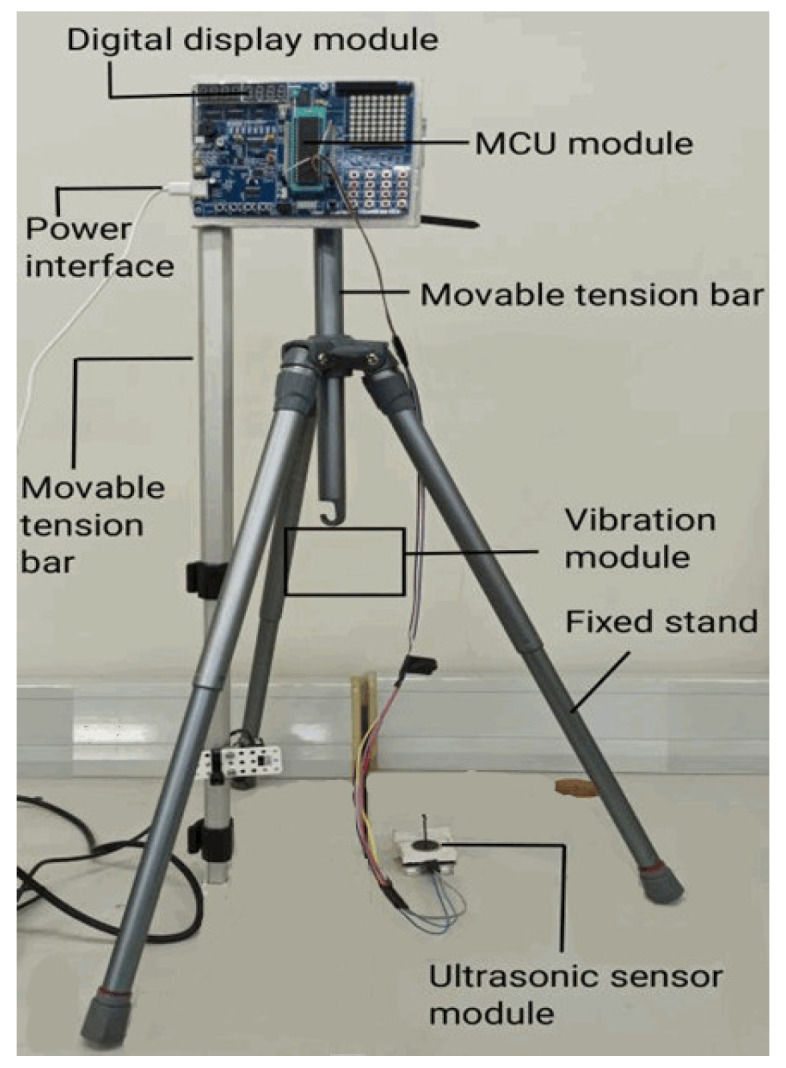
Photo of our ultrasonic testing instrument (ultrasonic sensor module was fixed together with the vibration module during use).

Our ultrasonic testing instrument consisted of a single-chip microcomputer (MCU) module (Model: 51 HC-SR04), a digital display module (digital tube), and an integrated ultrasonic sensor module (fixed together with the vibration module during use; referred to as the ultrasonic ranging module in context). The vibration change of the vibration module was measured by the ultrasonic sensor module, and the values of the measured distance parameter were displayed on the digital tube in real time. The photo of our ultrasonic testing instrument is shown in Figure 1.

#### 2.2.1. Single-Chip MCU Module

**Figure 2:**
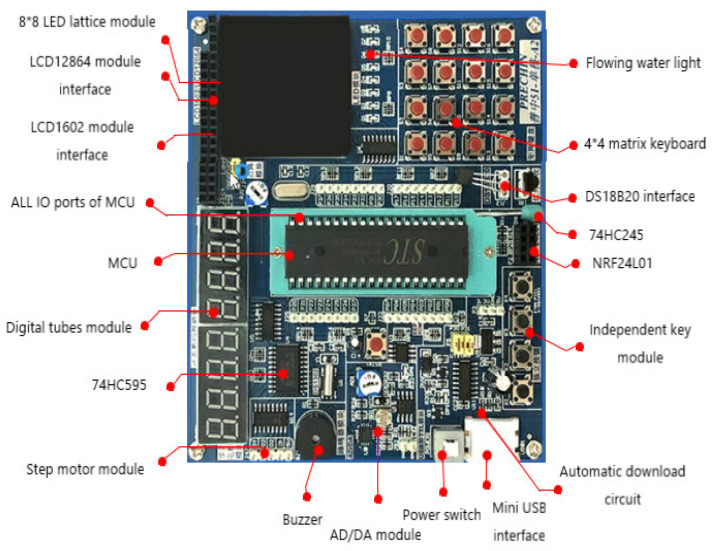
Interface of the single-chip MCU module.

The single-chip MCU module is shown in Figure 2. The function and name of each component are marked in the figure.

#### 2.2.2. Digital Display Module

**Figure 3:**
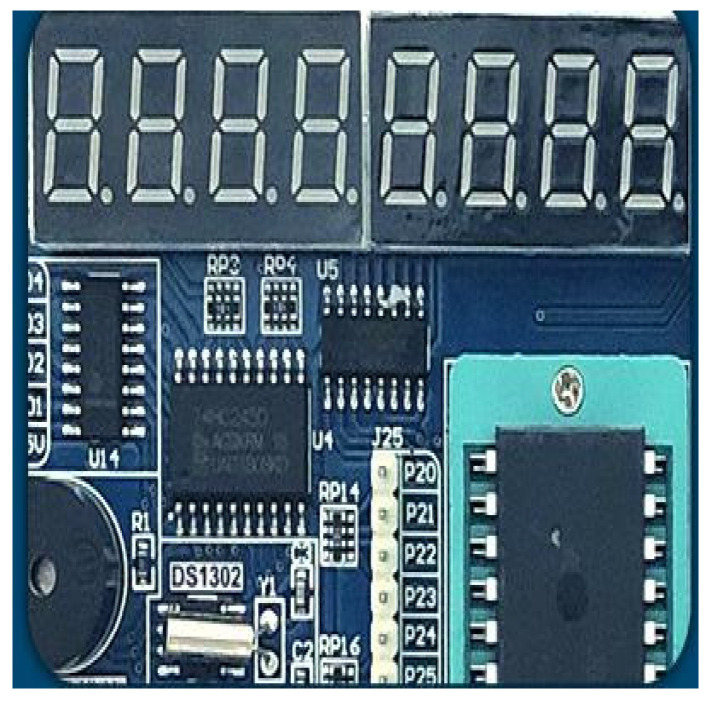
Photo of digital display module with an 8-digit tube.

As shown in Figure 3, the digital display module involved an 8-digit tube. It displayed the measurement data in real time. Using programming techniques, effective data with different numbers of digits can be implemented in the digital display module.

#### 2.2.3. Ultrasonic Sensor Module

**Figure 4:**
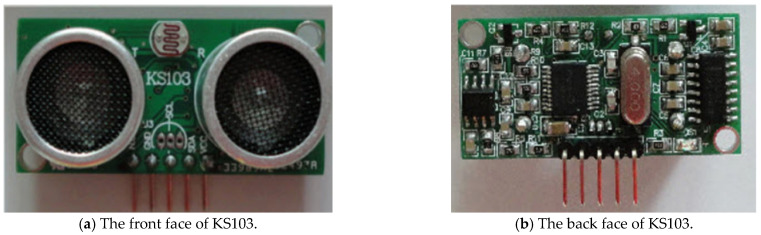
Photo of ultrasonic sensor module KS103.

As shown in Figure 4, the ultrasonic sensor module used an I2C or TTL serial port interface to communicate with the host and output the distance value in the millimeter order of magnitude. The range detection with real-time temperature compensation had high detection accuracy. Moreover, it could transmit multi-range detection instructions to meet the requirements of short-distance detection, along with quickly and precisely measuring the temperature and ultrasonic intensity.

### 2.3. Physical Principle of Ultrasonic Ranging

Ultrasonic waves are generated by the vibration of a transducer wafer under voltage excitation. When an ultrasonic wave hits an impurity or the interface of an obstacle, either it will produce a significant reflection to form an echo or the Doppler effect will occur if the wave meets a moving object.

**Figure 5:**
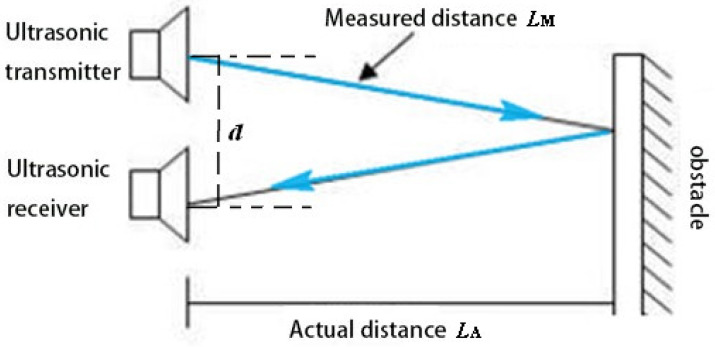
Physical principle of ultrasonic ranging.

Our instrument adopted dual probes: one was the transmitter, and the other was the receiver, as shown in Figure 4a and Figure 5. The distance between the transmitter and receiver is *d.* The relationship between the measured distance *L*_M_ and actual distance *L*_A_ is $L_{M}=\sqrt{L_{A}^{2}+\frac{d^{2}}{4}}$. When the value of *L*_A_ changes, the value of *L*_M_ can be displayed in real time on the digital display module through the measurement of the Doppler frequency shift by the ultrasonic ranging module.

## 3. Measuring Linear Springs’ Stiffness Factors

**Figure 6:**
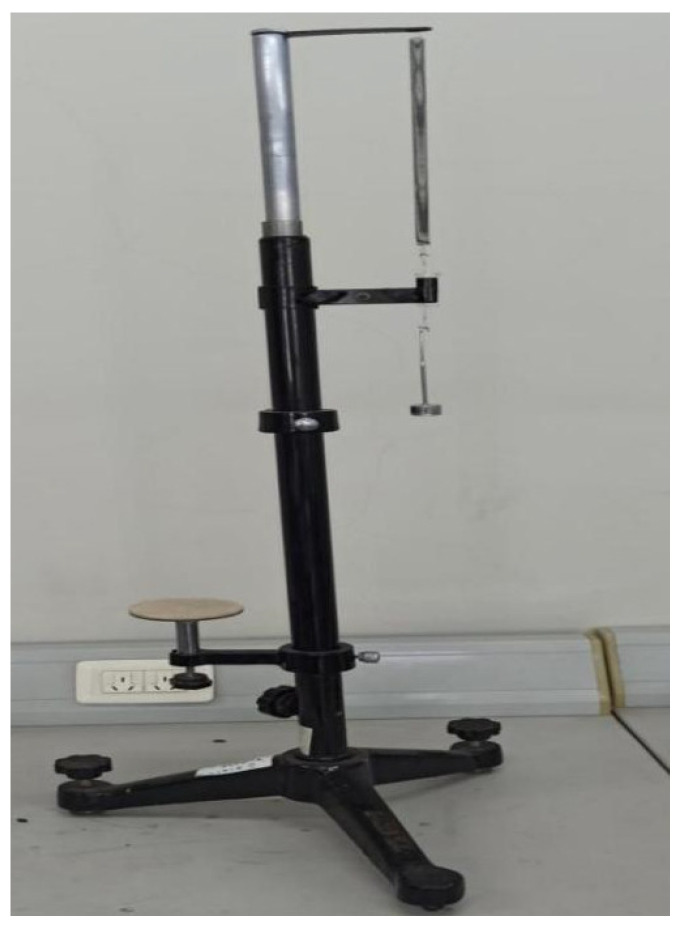
The stiffness factor *k* of the spring is measured with the Jolly’s scale.

**Figure 7:**
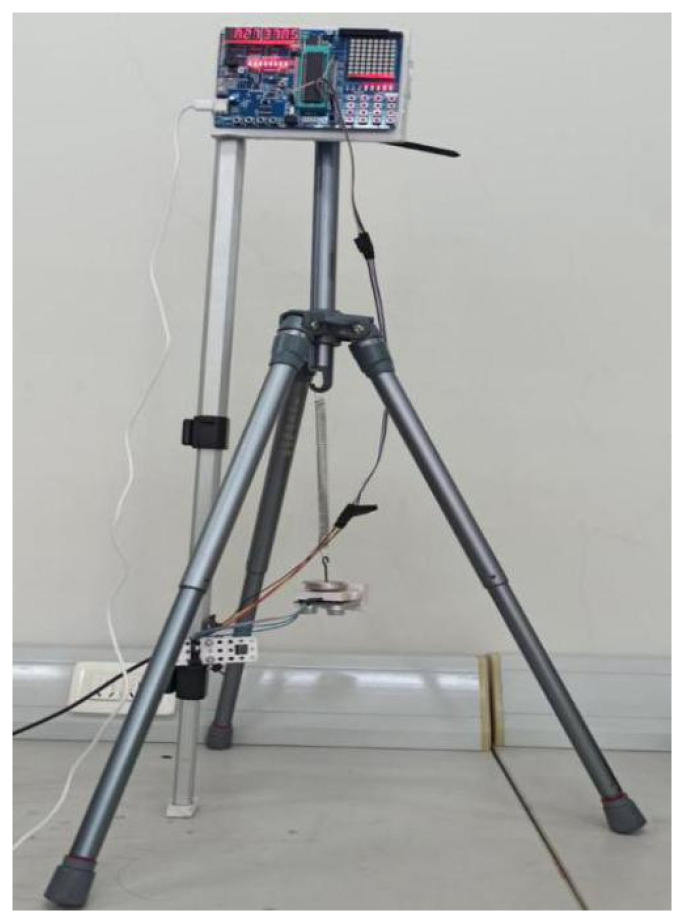
The stiffness factor *k* of the spring is measured by our ultrasonic testing instrument.

As shown in Figure 6 and Figure 7, we selected three linear springs with different stiffness factors and measured them with the traditional Jolly’s scale and our ultrasonic testing instrument, respectively. (Note: The three legs of our instrument were strictly locked to prevent sliding.) In the experiments, we added a fixed mass (5.05 g) to the lower end of each spring each time in turn, and we recorded the readouts of the two instruments when their states were stable. The readout *l_i_* (*i* = 0, 1, 2, 3, 4, 5) on the Jolly’s scale is a series of indicators of the vernier caliper when the scale realizes “aligning the three lines”, while the readout *l_i_* (*i* = 0, 1, 2, 3, 4, 5) on the ultrasonic sensor is a series of distance values between the ranging module and the horizontal bottom plate after the vibration module is stable and stationary. Our instrument automatically showed these values with the digital display module, which ruled out artificial accidental errors. $\bar{\Delta l}=\left|\left[\left(l_{3}-l_{0}\right)+\left(l_{4}-l_{1}\right)+\left(l_{5}-l_{2}\right)\right]/3\right|$ is the average difference of spring’s elongation. The measured and processed results are listed in Table 1. Among them, $k=\frac{\Delta m\Delta g}{\bar{\Delta l}}=\frac{\left(15.15\;–\;0.00\right)\times 10^{-3}\times 9.8}{\bar{\Delta l}\times 10^{-2}}$, and the standard deviation of the deformation is $\sqrt{\frac{{\left[\left(l_{3}-l_{0}\right)-\bar{\Delta l}\right]}^{2}+{\left[\left(l_{4}-l_{1}\right)-\bar{\Delta l}\right]}^{2}+{\left[\left(l_{5}-l_{2}\right)-\bar{\Delta l}\right]}^{2}}{3-1}}$. The relative error of *k* is $\left|k-k_{0}\right|/k_{0}\times 100\%$. True value *k*_0_ is the linear spring’s stiffness factor provided by the factory and measured with a dynamometer, according to Hooke’s law. We bought springs with different stiffness factors from the factory, and the manufacturer marked the values of the stiffness factors on the outer package. We referred to these as the true values. In Chinese university-level physics experiment textbooks, the term ”true value” is a default regulation.

For the expression of *k*, the combinations of *l*_3_ and *l*_0_, *l*_4_ and *l_1_*, and *l*_5_ and *l*_2_ correspond to 15.15 g and 0.00 g, 20.20 g and 5.05 g, and 25.25 g and 10.10 g, respectively. Thus, Δ*m* = 15.15 − 0.00 = 20.20 − 5.05 = 25.25 − 10.10 = 15.15 (g), which means that it concerns a series of measures with the same weight.

In general, the smaller the value of the standard deviation is, the smaller the deviation of the measurement value is, and the more accurate the measurement is. The smaller the value of the relative error of *k* is, the closer the measurement value is to the true one.

**Figure 8:**
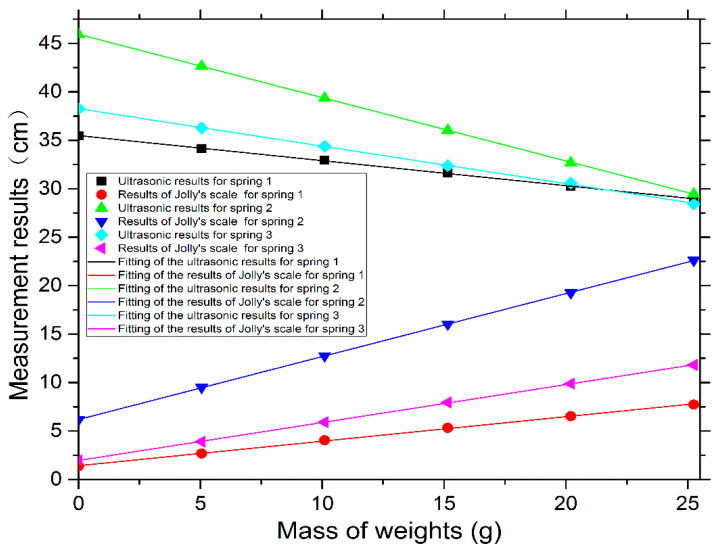
(Color online) Comparison between the measurement results of the three springs using our instrument and Jolly’s scale, accompanied by their respective fitting straight lines.

Table 1 shows that the stiffness factors of the same group of three springs measured by our ultrasonic testing instrument and Jolly’s scale were very close, and the standard deviations of the deformation indications were very small. Clearly, the standard deviations of the deformation measured by our ultrasonic testing instrument were much smaller than those of Jolly’s scale, demonstrating the accuracy of our ultrasonic testing instrument’s results. In addition, we know their true values. The relative errors between the measured results by our ultrasonic testing instrument and the true values were 0.26%, 0.00%, and 0.00%, respectively, better than those of Jolly’s scale, which were 1.8%, 0.67%, and 1.6%, respectively. Using the software Origin Pro 2015 to map, Figure 8 shows the comparison between the measurement results from our instrument and Jolly’s scale accompanied by their respective fitting straight lines. It is known from the software that the standard deviations of the slopes of the fitting straight lines obtained are all within 0.004, exhibiting perfect linearity in the fitting diagram and high accuracy in the measurement results. In summary, the results in Table 1 and Figure 8 show clearly that our ultrasonic testing instrument can accurately measure the stiffness factor of springs, that it can fully realize the performance of the traditional Jolly’s scale, and that its measurement results are more accurate than those of Jolly’s scale.

In general, both temperature and humidity have effects on ultrasonic waves [29,30]. To verify the sensitivity and stability of our ultrasonic testing instrument, we measured the stiffness factors of each spring at different times on the same day (under different temperatures and humidity levels). The experiment and processed results are shown in Table 2 and Table 3, respectively.

**Figure 9:**
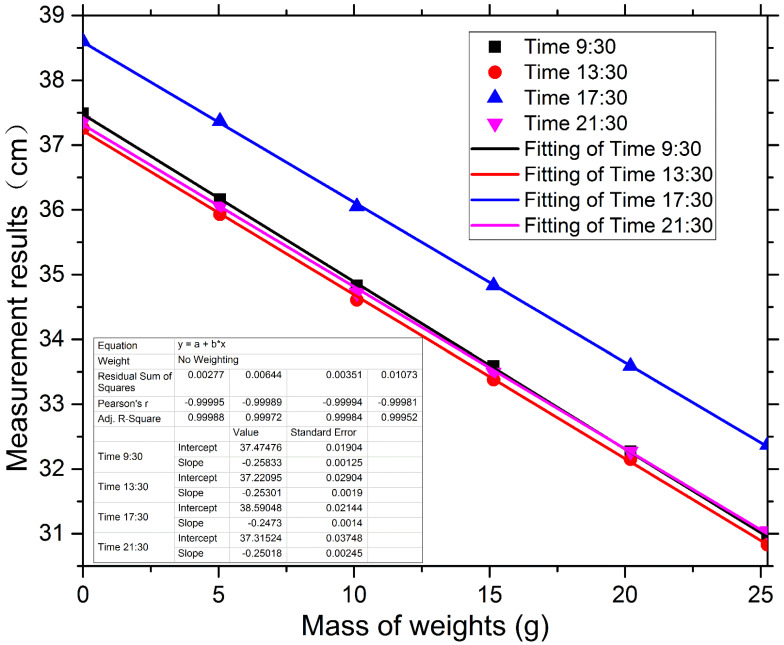
(Color online) Measurement results of each spring using our instrument at different times on the same day under different temperatures and humidity levels, accompanied by their respective fitting lines.

Table 2 shows the comparison of each spring’s stiffness factor with our ultrasonic testing instrument under different temperatures and humidity levels. To express these data intuitively, we converted them into Figure 9, which shows perfect linearity in the fitting diagram and high accuracy in the measurement results. The slopes of these four fitting lines are exactly equal, and they almost coincide with the exception of the line representing Time 17:30. For the line representing Time 17:30, we consciously increased the initial height of the *l*_0_ to test the sensitivity of our instrument. As expected, the slope of the fitting line formed by subsequent measurements was perfectly consistent with the slopes of the other three. This showed that our instrument was sensitive to the initial value.

Using the values of *k_i_* and the stiffness factor of each spring at different times on the same day, obtained from a series of measures with the same weight, we performed the uncertainty analysis based on the data in Table 2 and Table 3 as follows: Our instrument’s instrument error value was $\Delta q=k_{0}-k_{i,min}=3.80-3.791127=0.008873\;\left(N/m\right)$. The overall uncertainty is $u_{x}=\sqrt{\Delta q^{2}+{\bar{S}}^{2}}=\sqrt{0.008873^{2}+0.07^{2}}\approx$ 0.07 (N/m). Considering the overall uncertainty, $k_{i}=\bar{k}\pm u_{x}=3.79\pm 0.07\;\left(N/m\right)$. This meant that the probability of *k_i_* was high in the interval (3.72, 3.86), and the probability of true value being outside the interval was small.

Table 3 showed that the mean value of the measured stiffness factors of each spring using our ultrasonic testing instrument under different temperatures and humidity levels was 3.79 N/m. We know the true value of this spring is 3.80 N/m, and the following data are calculated on the true value. The average standard deviation of the measured stiffness factors was 0.06 N/m, and the relative average standard deviation was 1.6% (<5.0%, confidence interval). The relative error of *k_i_* was 0.26%, and the values of the standard errors are all within the range of 0.00–0.05 N/m. All these data indicate that the difference extent between the stiffness factors measured by our instrument and the true value 3.80 N/m was negligible and reflected the high precision of the measurement results. In summary, our ultrasonic testing instrument measured the linear springs’ stiffness factors under diverse temperatures and humidity levels, with high data reliability and perfect stability.

## 4. Conclusions

We designed an ultrasonic testing instrument that used ultrasonic waves to measure the stiffness factors of linear springs. We measured the stiffness factors of three different linear springs with Jolly’s scale and our instrument. By comparison, we found that our instrument was more convenient to operate than Jolly’s scale. We verified that our instrument had the advantages of accurate results with little error, perfect sensitivity, and stable performance in measuring the linear springs’ stiffness factors under diverse temperatures and humidity levels, which precisely surpassed the performance of Jolly’s scale. In addition, our instrument can also be exploited to measure the surface tension and surface tension coefficients of liquids, Young’s modulus of a wire, tensile strength of a paper, tiny deformation of objects, flexibility of a wool wire, and other quantitative measurements.

## Figures and Tables

**Table 1:** Comparison table of the measurement results of the three springs’ stiffness factors.

	Mass of Weights (g)	0.00	5.05	10.10	15.15	20.20	25.25				
Group	Measuring Tool	$l_{0}$ (cm)	$l_{1}$ (cm)	$l_{2}$ (cm)	$l_{3}$ (cm)	$l_{4}$ (cm)	$l_{5}$ (cm)	$\bar{\Delta l}\;(\mathrm{cm})$	*k* (N/m)	Standard Deviation of the Deformation (cm)	Relative Error of *k* (%)
Spring 1	Jolly’s scale	1.37	2.67	4.05	5.33	6.54	7.73	3.84	3.87	0.14	1.8
	Ultrasonic sensor	35.45	34.16	32.94	31.61	30.26	28.92	3.92	3.79	0.09	0.26
	True value *k*_0_	/	/	/	/	/	/	/	3.80	/	/
Spring 2	Jolly’s scale	6.16	9.50	12.76	16.03	19.27	22.61	9.83	1.51	0.05	0.67
	Ultrasonic sensor	45.92	42.64	39.37	36.04	32.69	29.47	9.91	1.50	0.04	0.00
	True value *k*_0_	/	/	/	/	/	/	/	1.50	/	/
Spring 3	Jolly’s scale	1.98	3.90	5.91	7.84	9.78	11.72	5.85	2.54	0.04	1.6
	Ultrasonic sensor	38.26	36.27	34.38	32.32	30.34	28.46	5.93	2.50	0.01	0.00
	True value *k*_0_	/	/	/	/	/	/	/	2.50	/	/

**Table 2:** Measurement results of each spring using our ultrasonic testing instrument at different times on the same day under different temperatures and humidity levels.

			Mass of Weights (g)	0.00	5.05	10.10	15.15	20.20	25.25	
Date	Time	Temperature (°C)	Humidity (%)	$l_{0}\left(cm\right)$	$l_{1}\left(cm\right)$	$l_{2}\left(cm\right)$	$l_{3}\left(cm\right)$	$l_{4}\left(cm\right)$	$l_{5}\left(cm\right)$	*k_i_* (N/m)
On the same day	9:30	17.2	74	37.51	36.16	34.83	33.55	32.27	30.94	3.794220
	13:30	18.1	71	37.29	35.96	34.71	33.34	32.1	30.77	3.791127
	17:30	16.4	79	38.68	37.47	36.15	34.73	33.49	32.33	3.791928
	21:30	15.2	80	37.41	36.14	34.82	33.44	32.21	30.97	3.791342
			True value *k*_0_	/	/	/	/	/	/	3.80

**Table 3:** Statistics of the stiffness factors of the same spring under different temperatures and humidity levels.

Temperature (°C)	Humidity (%)	*k_i_* (N/m)	$\bar{k}$	$S_{i}\;(N/m)$	$\bar{S}\;(N/m)$	ReAD (%)	$E_{X}(\%)$	SE (N/m)
17.2	74	3.794220	3.79	0.04	0.07	1.6	0.26	0.03
18.1	71	3.791127		0.05				0.03
16.4	79	3.791928		0.09				0.05
15.2	80	3.791342		0.07				0.04
True value *k*_0_		3.80	/	/	/	/	/	/

Note: Each stiffness factor of each spring at different times on the same day is $k_{i}=\frac{\Delta m\cdot g}{\Delta l_{i}}/3=\left[\frac{\Delta m\times 10^{-3}\times 9.8}{\left(l_{0}-l_{3}\right)\times 10^{-2}}+\frac{\Delta m\times 10^{-3}\times 9.8}{\left(l_{1}-l_{4}\right)\times 10^{-2}}+\frac{\Delta m\times 10^{-3}\times 9.8}{\left(l_{2}-l_{5}\right)\times 10^{-2}}\right]/3;$ Δm=15.15 (g). The mean value of k_i_ is $\;\bar{k}=\frac{1}{4}\sum_{i=1}^{4}k_{i}$. The standard deviation of *k_i_* is $S_{i}=\sqrt{\frac{\sum_{i=1}^{4}{\left(k_{i-}\bar{k}\right)}^{2}}{4-1}}$. The average standard deviation of *k_i_* is $\bar{S}=\frac{S_{1}+S_{2}+S_{3}+S_{4}}{4}$. The relative average standard deviation of *k_i_* is ReAD = $\bar{S}$/$\bar{k}$. The relative error of *k_i_* is $\;E_{X}=\frac{\left|\bar{k}-k0\right|}{\;k0}\times 100\%$. The standard error of *k_i_* is SE = $S_{i}/\sqrt{3}$.

## Data Availability

We provide detailed data supporting reported results can be found in all the Tables and Figures in this paper.
